# Treatment of Recurrent Cesarean Scar Pregnancy With Oral Mifepristone, Systemic Methotrexate, and Ultrasound-Guided Suction Dilation and Curettage

**DOI:** 10.7759/cureus.36200

**Published:** 2023-03-15

**Authors:** Megan Masten, Meredith Alston

**Affiliations:** 1 Obstetrics and Gynecology, Denver Health and Hospitals, Denver, USA; 2 Obstetrics and Gynecology, Saint Joseph Hospital, Denver, USA

**Keywords:** d&c, mifepristone, methotrexate, recurrent cesarean scar pregnancy, cesarean scar pregnancy

## Abstract

Cesarean scar pregnancy (CSP), or pregnancies with implantation in a prior cesarean section scar, are rare but may be becoming more common with an increase in cesarean section deliveries. History of prior CSP may also increase the risk for recurrent CSP. Several treatment options and combinations of treatment options for CSP have been described in the literature. Although the optimal treatment is unclear, the Society of Maternal-Fetal Medicine published recommendation guidelines, which include recommendations for the treatment/termination of CSP pregnancies. Treatment of CSP is recommended with operative resection, ultrasound-guided suction dilation and curettage (D&C), or intragestational methotrexate with or without treatment modalities. This is a case report of a patient with recurrent CSP. Her first CSP was incorrectly diagnosed as an incomplete abortion after unsuccessful treatment with misoprostol alone and ultimately was successfully treated with systemic methotrexate. Her second CSP is the basis of this case report and was successfully treated with oral mifepristone and systemic methotrexate (50 milligrams/meter^2^) before an ultrasound-guided suction D&C at 10 weeks 1 day gestational age. The combination of mifepristone, systemic methotrexate, and suction D&C under ultrasound guidance as a treatment for recurrent CSP has not previously been described in published literature.

## Introduction

Cesarean scar pregnancy (CSP) is rare. Previous estimates ranged from 1:1,800 to 1:2,656 of overall pregnancies from the early 2000s but the incidence of CSP may be becoming more common with the increasing rate of cesarean section deliveries [1-5]. A study from 2011 based at a large tertiary care hospital in Israel estimated the incidence of cesarean scar pregnancy as one per 3,000 for the general obstetric population and one per 531 among those who had undergone at least one cesarean delivery [5]. In that study, cesarean scar pregnancy constituted 4.2% of ectopic pregnancies [5].

CSP by definition is a pregnancy in a prior cesarean scar and has risks for severe maternal morbidity such as uterine rupture, hemorrhage, or live births complicated by placenta accreta spectrum (PAS), hemorrhage, and/or risk of cesarean hysterectomy [1-7]. Additionally, it’s likely that patients with a prior CSP are at higher risk of recurrence [8,9]. Published rates for the recurrence of CSP have been published between 17.6% and 34.3% [8,9].

Expectant management is not recommended by the Society of Maternal-Fetal Medicine (SMFM) when the diagnosis of CSP is made [2]. Multiple treatments and combinations of treatments for CSP have been reported in the literature, but the optimal treatment is unclear [2]. Per the SMFM Consult series, recommendations for treatment of CSP pregnancies include active management with operative resection, ultrasound-guided suction dilation and curettage (D&C), or intragestational methotrexate with or without other modalities. SMFM recommends against systemic methotrexate alone [2]. Mifepristone, which is used widely in conjunction with misoprostol for abortion management, is another medication that may be used in CSP in conjunction with other methods, although it was not described in the SMFM Consult series [10-14].

Despite having information on effective treatment options for CSP, there is no consensus regarding guidelines for treatment for recurrent CSP [8,9]. Although mifepristone, methotrexate, and ultrasound-guided suction D&C have been used for CSP successfully, there are no published reports of this regimen for recurrent CSP [8].

## Case presentation

**Figure 1:**
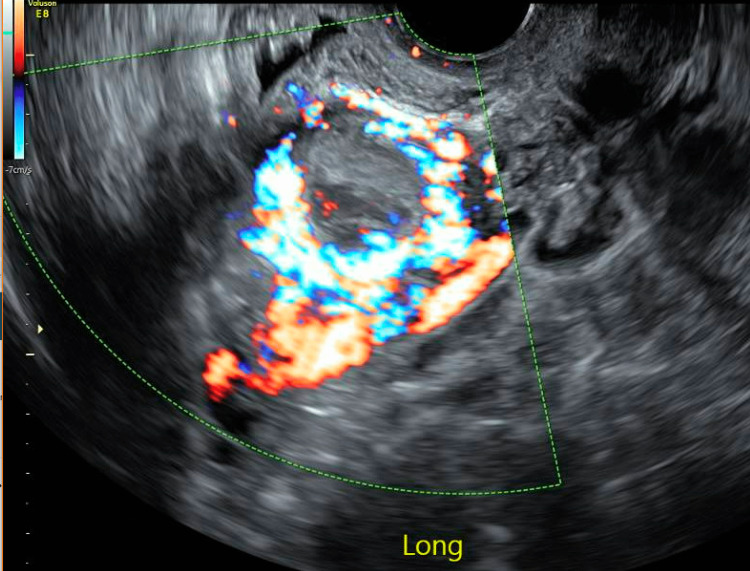
Retained products of conception with cesarean scar pregnancy

The patient was a 30-year-old gravida 7 para 3-0-3-3 who had three prior cesarean sections. She had her third cesarean section four years prior to her re-presentation and was found to have a uterine window noted just superior to her bladder. She had another pregnancy two years later, and with her first ultrasound, a crown-rump length was noted measuring six weeks gestational age. She underwent a viability scan two weeks later, where she had an incorrectly diagnosed incomplete abortion based on loss of the fetal pole and yolk sac. She initially opted for expectant management. When she did not pass the pregnancy on her own, she opted for medical management with misoprostol. She experienced bleeding after misoprostol and had a bedside ultrasound in the clinic, which was concerning for retained products of conception. She then had a subsequent formal ultrasound concerning for CSP with invasive placentation extending to her bladder, with concern for posterior bladder wall involvement (Figure 1). At that time, there was no gestational sac, yolk sac, or fetal pole noted.

She was offered multiple management options at that time including medical management with systemic versus local methotrexate and surgical/procedural management including hysterectomy or uterine artery embolization. She opted for systemic methotrexate at 50 milligrams/meter^2^ (mg/m^2^). At that time (day 0), her beta-human chorionic gonadotropin (bhCG) was 244 milli-international units per milliliter of blood (mIu/mL). A repeat ultrasound two weeks later showed a decrease in size of retained products of conception (area measuring 4 by 4.5 centimeters originally to 3 by 3.5 centimeters). Her bhCG was followed over the next several weeks and after about four weeks, the patient’s bhCG downtrended to negative (4 mIu/mL). She was counseled against future pregnancy.

**Figure 2:**
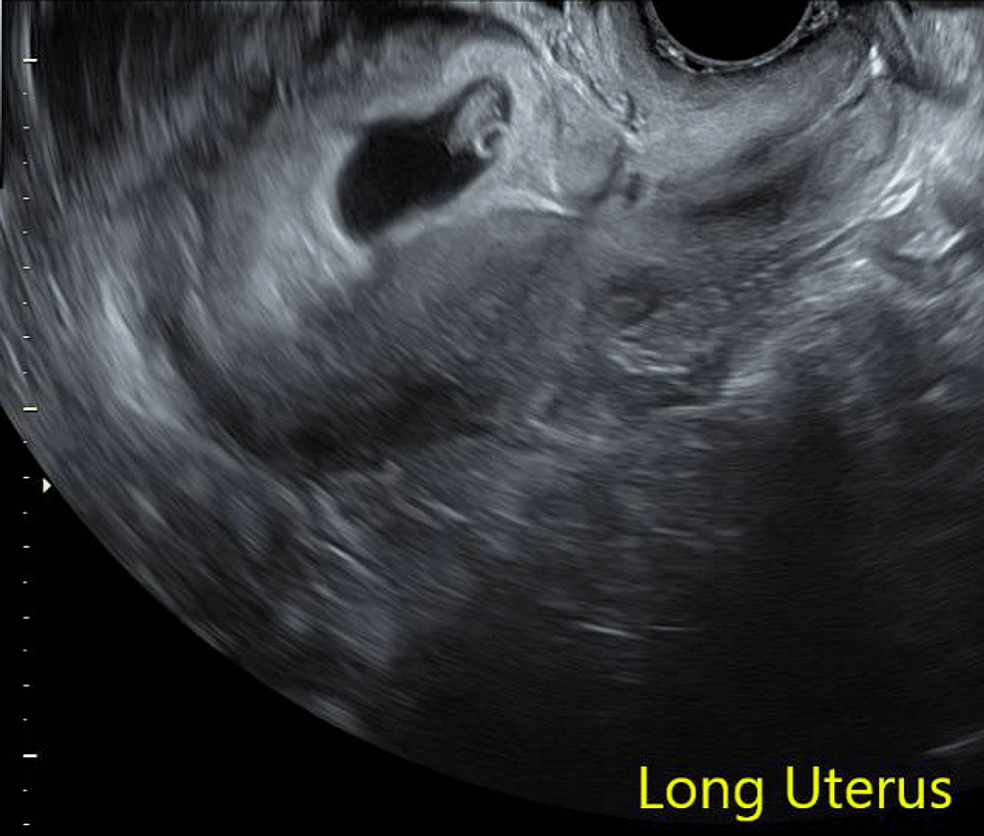
Recurrent cesarean scar pregnancy at seven weeks gestational age

The basis of this case report focuses on her most recent pregnancy. The patient presented for amenorrhea and was found to have a positive urine pregnancy test. The pregnancy was unplanned but desired. At seven weeks gestational age, she had her first transvaginal ultrasound, which showed a pregnancy concordant with her last menstrual period with cardiac activity and concern for implantation in her cesarean scar (Figure 2). Of note, there was an additional cesarean scar visualized inferior to the scar where the pregnancy is implanted.

She was counseled about CSP and PAS and was recommended treatment to terminate the pregnancy. She was offered operative management with suction D&C, cervical ripening balloon placement, or gravid hysterectomy, and she was offered systemic methotrexate to be used with any of the operative options. She desired a repeat ultrasound for verification of CSP given this pregnancy was highly desired if not a CSP, and this was done at nine weeks. With her repeat ultrasound, she was again noted to have a pregnancy implanted in her prior cesarean scar and had trophoblastic tissue extending to within 1.2 millimeters (mm) of the anterior uterine wall, without evidence of invasion into bladder. Again, this was discussed with the patient, and she wished to proceed with treatment at that time. Although she did not plan future fertility, she strongly desired to avoid a hysterectomy. She was recommended a combination of medical and surgical management, which was based on operating room availability, desire for cervical preparation, and preference to end fetal cardiac activity prior to the procedure if possible.

At 9 weeks 4 days gestational age, she received 200 milligrams (mg) of oral mifepristone and systemic methotrexate with 50 mg/m2 of methotrexate given intramuscularly. She was scheduled for her suction D&C in the operating room to occur four days later, due to block time availability and desire for the procedure to happen during the weekday with additional staff available. Four days later (at 10 weeks 1 day gestational age) she presented for suction D&C in the operating room under ultrasound guidance. After arrival, she started developing pain and cramping, although symptoms were stable until the time of her scheduled procedure and her procedure did not need to be performed more urgently or emergently. Fetal cardiac activity was noted on bedside ultrasound at the time of D&C. Her suction D&C was uncomplicated and estimated blood loss was 75 milliliters (mL). She did receive one dose of intramuscular methergine intraoperatively due to initial brisk vaginal bleeding after removal of the gestational sac and she received a 24-hour course of oral methergine. She was monitored in the hospital overnight with minimal bleeding, and she was discharged on postoperative day 1. Given that the pregnancy had been removed under ultrasound guidance, bhCG was not followed. There were no complications. She received depot medroxyprogesterone acetate initially and subsequently underwent a laparoscopic salpingectomy for permanent contraception several months later.

## Discussion

This was an example of recurrent CSP, which was successfully treated with mifepristone, methotrexate, and suction D&C at 10 weeks gestational age. Aspects of this case that are unique include pre-treatment with mifepristone and methotrexate before suction D&C as well as treatment of a recurrent CSP.

Although several case studies have published treatment courses that include mifepristone for live pregnancies with CSP [10-12], there is one published retrospective analysis that includes a treatment arm including treatment with mifepristone, systemic methotrexate, and operative management which is most similar to the current case report [10]. Yu et al. examined three different treatment arms in a retrospective analysis: mifepristone and operative management versus methotrexate and operative management versus mifepristone with methotrexate and operative management [10]. In this case report, treatment included oral mifepristone, systemic methotrexate, and embryo removal with suction D&C. Differences between the retrospective analysis and the current case report include that in the retrospective analysis, methotrexate was given in oral and/or intramuscular routes, and operative management included suction D&C, hysteroscopic resection, or hysterotomy by laparotomy. Additionally, the retrospective analysis did not include participants with recurrent CSP. The study team ultimately deemed that mifepristone and methotrexate combined with embryo removal is a reliable way to treat CSP.

Given that rates of recurrence of CSP have been published to be as high as 34.3%, information about effective treatment options for CSP is important [9]. The current case report is an example of recurrent CSP, which was successfully treated with oral mifepristone, a single dose of systemic methotrexate, and suction D&C in the operating room to follow. Given the success, this could be a treatment for future patients in this scenario, at least to a gestational age of 10 weeks 1 day.

## Conclusions

In the situation where a patient has a history of prior CSP and has a recurrence of CSP, treatment could be considered with mifepristone (200mg once orally) and systemic methotrexate (50 mg/m^2^ once intramuscularly) before a suction D&C in the operating room, given that the current case report includes this treatment for a recurrent CSP at 10 weeks 1 day gestational age. Future research endeavors could include a randomized control trial to compare treatment with mifepristone, systemic methotrexate, and suction D&C to either mifepristone or systemic methotrexate with suction D&C and to suction D&C alone.
